# Balloon traction for lumen-apposing metal stent placement for walled-off necrosis: balloon adventure in WONderland

**DOI:** 10.1055/a-2325-2070

**Published:** 2024-06-05

**Authors:** Kohei Kurihara, Tomotaka Saito, Tsuyoshi Hamada, Yousuke Nakai, Mitsuhiro Fujishiro

**Affiliations:** 1 Department of Gastroenterology, Graduate School of Medicine, The University of Tokyo, Tokyo, Japan; 2 Department of Hepato-Biliary-Pancreatic Medicine, The Cancer Institute Hospital of Japanese Foundation for Cancer Research, Tokyo, Japan; 3 Department of Endoscopy and Endoscopic Surgery, The University of Tokyo Hospital, Tokyo, Japan

The electrocautery-enhanced lumen-apposing metal stent (LAMS) has enabled simple direct puncture of walled-off necrosis (WON) under endosonographic guidance 1 2 3 . However, the two-step approach (e.g. use of a guidewire and balloon dilation 4 ) may be required for solid WON lesions. In the current case, part of the gastric wall moved away from the echoendoscope during stent deployment and was successfully pulled back using an inflated balloon dilator ( Video 1 ).

The balloon traction technique for endoscopic ultrasound-guided placement of a lumen-apposing metal stent for solid walled-off necrosis.Video 1

A 53-year-old man was hospitalized for infectious WON located near the pancreatic head ( Fig. 1 ). Given the insufficient internal liquefaction, we decided to perform wire-guided LAMS placement. Following puncture from the greater curvature of the lower gastric body with a 19-G needle and insertion of a 0.025-inch guidewire, we attempted to advance the delivery catheter of a 15-mm-wide LAMS (Hot AXIOS; Boston Scientific Japan, Tokyo, Japan). However, entry of the delivery catheter into the WON was impossible due to recoil, resulting in an unstable echoendoscope position as well as a gap between the lesion and the echoendoscope ( Fig. 2 **a** ). Using an 8-mm balloon dilator (ZARA; Kaneka, Tokyo, Japan), we dilated along the puncture tract and within the internal contents. We then pulled the inflated balloon to the gastric side, which successfully brought the WON wall against the echoendoscope probe ( Fig. 2 **b** ). Finally, the LAMS was placed readily ( Fig. 2 **c** ), followed by placement of a 7-Fr nasal catheter for irrigation. Post-procedural computed tomography confirmed the appropriate stent location with no signs of leakage of the intracystic contents ( Fig. 2 **d** ).

**Fig. 1:**
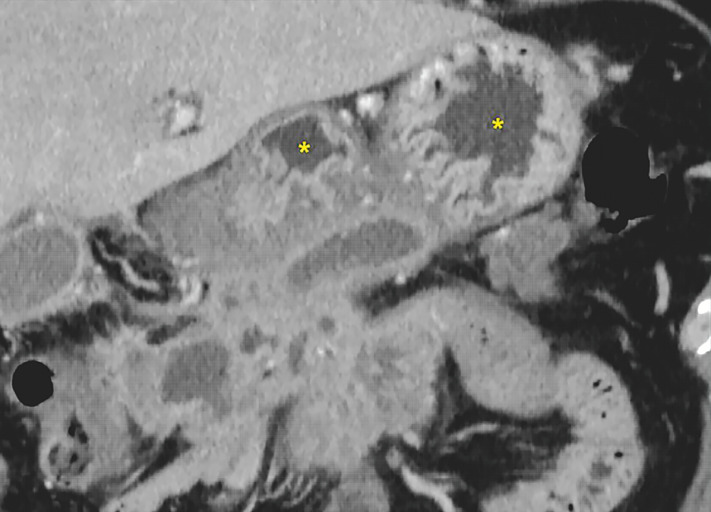
Computed tomography delineating a large walled-off necrotic lesion with a maximum diameter of 13 cm and edematous thickening of the gastric wall (asterisks).

**Fig. 2:**
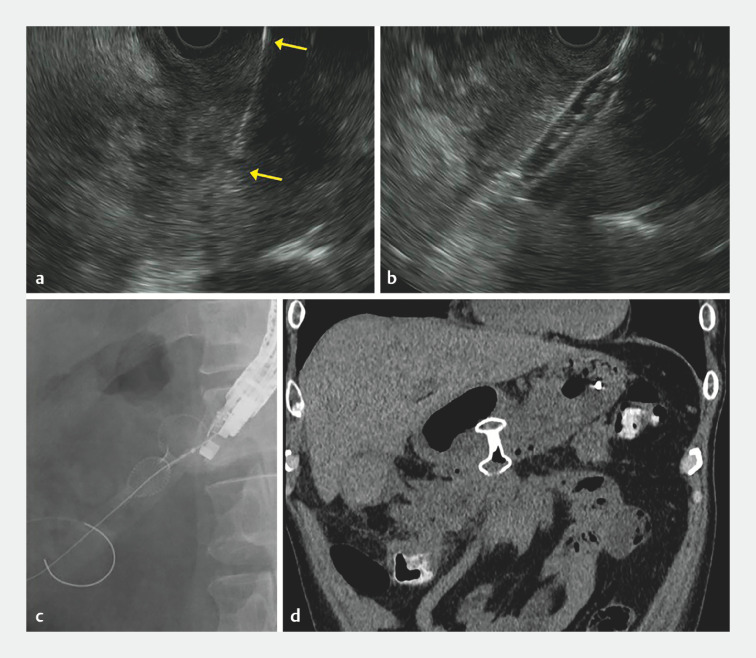
Endoscopic ultrasound-guided placement of a lumen-apposing metal stent (LAMS) for walled-off necrosis (WON) using the balloon traction technique. **a** Endosonographic view showing a gap between the WON and the tip of an echoendoscope (space between arrows), with no clear visualization of the WON. **b** Endosonographic view showing an inflated balloon dilater pulling the WON against the gastric wall. **c** Subsequent successful placement of the LAMS. **d** Computed tomography demonstrating appropriate positioning of the LAMS with no leakage of intracystic contents.

In cases of WON with low-level liquefication, the short delivery system of the LAMS may hamper appropriate positioning of its distal flange within the lesion 5 . The “balloon traction” technique may be a salvage procedure when the echoendoscope position becomes unstable due to recoil during insertion of the LAMS delivery catheter.

Endoscopy_UCTN_Code_TTT_1AS_2AD

## Notes

Endoscopy E-Videos https://eref.thieme.de/e-videos
*E-Videos* is an open access online section of the journal *Endoscopy* , reporting on interesting cases and new techniques in gastroenterological endoscopy. All papers include a high-quality video and are published with a Creative Commons CC-BY license. Endoscopy E-Videos qualify for HINARI discounts and waivers and eligibility is automatically checked during the submission process. We grant 100% waivers to articles whose corresponding authors are based in Group A countries and 50% waivers to those who are based in Group B countries as classified by Research4Life (see: https://www.research4life.org/access/eligibility/ ). This section has its own submission website at https://mc.manuscriptcentral.com/e-videos .
